# The Role of Lactate-Mediated Metabolic Coupling between Astrocytes and Neurons in Long-Term Memory Formation

**DOI:** 10.3389/fnint.2016.00010

**Published:** 2016-03-03

**Authors:** Michael Q. Steinman, Virginia Gao, Cristina M. Alberini

**Affiliations:** Center for Neural Science, New York UniversityNew York, NY, USA

**Keywords:** astrocyte, neuron, learning, memory, lactate, glucose, glucose metabolism

## Abstract

Long-term memory formation, the ability to retain information over time about an experience, is a complex function that affects multiple behaviors, and is an integral part of an individual’s identity. In the last 50 years many scientists have focused their work on understanding the biological mechanisms underlying memory formation and processing. Molecular studies over the last three decades have mostly investigated, or given attention to, neuronal mechanisms. However, the brain is composed of different cell types that, by concerted actions, cooperate to mediate brain functions. Here, we consider some new insights that emerged from recent studies implicating astrocytic glycogen and glucose metabolisms, and particularly their coupling to neuronal functions via lactate, as an essential mechanism for long-term memory formation.

## Introduction

A newly formed memory is temporarily labile, and can become a long-term, stable memory if it undergoes a process known as *consolidation*. Memory consolidation is a fundamental, evolutionarily conserved process that occurs in different types of long-term memories and depends upon an initial phase of gene expression, which, if interrupted, results in amnesia (Davis and Squire, 1984; McGaugh, 2000; Alberini, 2009). At the cellular level, long-term memory has been studied using long-term potentiation (LTP) that produces activity-dependent increases in synaptic strength, generally evoked by high frequency stimulation (Malenka, 1994). Although not always fully corresponding, both long-term memory and LTP share the initial *de novo* gene expression requirement, involve the activation of similar molecular pathways such as MAPK, CaMKIIα, PKA and PKC (Martin et al., 1997), and are accompanied by synapse morphological modifications (Toni et al., 1999). Thus, memory consolidation has historically been considered a product of neuronal changes, and has been studied with a near total emphasis on neuronal responses.

However, particularly during the last 10 years, it has become increasingly better established that the glial cells known as astrocytes actively participate in complex brain functions, including cognitive ones, once attributed solely to neurons (Haydon and Nedergaard, 2015). Notably, the greater focus on astrocytes has brought new insights into non-neuronal contributions to learning and memory and synaptic plasticity. Both long-term memory and plasticity, in fact, require astrocytic transmission and processing of signals (Stehberg et al., 2012; Moraga-Amaro et al., 2014). Astrocytes also critically contribute to the morphological remodeling associated with synaptic plasticity, hence possibly functioning as spatial and temporal integrators of neural activity and plasticity (Bourne and Harris, 2008; De Pittà et al., 2015). In addition, synaptic plasticity and memory rely on astrocytic regulations of nutrient availability; one that is particularly well-studied is glucose entry into the brain and its subsequent metabolism (Nedergaard et al., 2003; Halassa and Haydon, 2010). In this review, we will discuss evidence and questions concerning how glucose and glycogen metabolism in the brain, via production of lactate, are implicated in long-term synaptic plasticity and memory formation.

## Brain Glucose and Glycogen Metabolism, and the Hypothesis of an astrocyte-Neuron Lactate Shuttle (ANLS)

The glucose that astrocytes import from systemic blood has traditionally been believed to serve as the foremost substrate of energy directly supplied to and used by neurons. This, however, is now highly debated (e.g., Chih et al., 2001; Pellerin and Magistretti, 2012). Several studies in the last decades, including ours focusing on memory formation (Suzuki et al., 2011), have supported the hypothesis that, at least in physiological conditions of high-energy demands, that may not be the case. These studies indicate that L-lactate might in fact be preferentially utilized in these conditions. Many excellent reviews report the debated issues and different views (Pellerin et al., 1998; Chih et al., 2001; Dienel and Hertz, 2001; Chih and Roberts, 2003; Dienel and Cruz, 2004; Kimelberg, 2004; Bonvento et al., 2005; Fillenz, 2005; Hertz and Dienel, 2005; Pellerin et al., 2007; Barros and Deitmer, 2010; Belanger et al., 2011; Pellerin and Magistretti, 2012; Barros, 2013; Schurr, 2014; Dienel, 2015; Magistretti and Allaman, 2015; Weber and Barros, 2015; Kasparov, 2016) and we refer to these for a comprehensive overview of the debates and questions that still remain to be addressed. Our data, collectively, are in agreement with many studies summarized and discussed by Schurr (2014) and the conclusion that L-lactate plays a critical role in the physiology of brain functions and that “*glycolysis, in cerebral and other tissues, is a singular pathway, in the presence or absence of oxygen, which begins with glucose as its substrate and terminates with the production of lactate as its main end product*”.

Pellerin and Magistretti (1994) proposed that electrophysiological activity links activated glutamatergic neurons and astrocytes through lactate, which is produced glycolytically by astrocytes and shuttled to neurons to be used as a significant source of energy (the astrocyte-neuron lactate shuttle or the ANLS; Pellerin and Magistretti, 2012). The ANLS hypothesis originally derived from findings that glutamate triggered uptake of labeled glucose and release of L-lactate by cultured astrocytes (Pellerin and Magistretti, 1994). In line with this hypothesis, earlier work demonstrated that L-lactate was sufficient to maintain synaptic activity in rat hippocampal slices (Schurr et al., 1988), and that during focal physiologic neural activity the consumption of glucose is non-oxidative (Fox et al., 1988). Fox and Raichle (1986) had previously described a focal physiological uncoupling between cerebral blood flow and oxidative metabolism upon somatosensory stimulation in humans; thus, it was subsequently proposed that the temporal mismatch between glucose uptake and oxygen consumption in the brain observed in positron emissions tomography (PET) scans reflects the fact that glucose is taken up by astrocytes and metabolized through aerobic glycolysis prior to use by neurons (Magistretti and Pellerin, 1999). According to this idea, glucose uptake does indeed occur as brain regions activate, though it may not occur primarily in neurons, but, rather, in astrocytes. This has been confirmed in rat barrel cortex after whisker stimulation using a real-time method for measuring the uptake of the fluorescent glucose analog 6-[N-(7-Nitrobenz-2-oxa-1,3-diazol-4-yl)amino]-6-Deoxyglucose (6-NBDG) in astrocytes and neurons *in vivo*. During resting conditions the 6-NBDG uptake in astrocytes and neurons is similar, but during intense neuronal activity, triggered by whisker stimulation, astrocytes increase their uptake, whereas neurons do not, suggesting that stimulation elicits glucose uptake primarily in astrocytes (Chuquet et al., 2010). In contrast with these findings, a recent study demonstrated that whisker stimulation in awake mice causes neurons to take up near-infrared 2-deoxyglucose analog at a higher rate than astrocytes (Lundgaard et al., 2015), and identified the neuron as the principal locus of glucose uptake. One important caveat of using glucose analogs is that a change in size and structure may lead to their uptake via endocytosis rather than transport (Tadi et al., 2015). As endosomal trafficking is highly specialized in neurons (Yap and Winckler, 2012) further studies are needed to sort out if and in which functional conditions astrocytes or neurons have differential glucose uptake. In agreement with preferential uptake of glucose in astrocytes, downregulation of the expression of glial glutamate transporters impairs the 2-deoxyglucose autoradiographic signal in barrel field following stimulation of the corresponding whisker, indicating that glutamate uptake into astrocytes is the trigger for glucose uptake by the brain parenchyma (Voutsinos-Porche et al., 2003).

In contrast, studies have reported a preferential uptake of glucose by neurons during periods of activity. For example Bak et al. (2009) showed that administration of NMDA elicits increased neuronal uptake of glucose but not L-lactate; however it has been suggested that the dose of NMDA used in these experiments is excitotoxic (Bouzier-Sore and Pellerin, 2013). Another study showed that following seizure, nerve terminals extracted from rats had increased hexokinase-mediated phosphorylation of glucose analog 2-fluoro-2-deoxy-D-glucose indicating neuronal glucose oxidation (Patel et al., 2014). It is important to point out, however, that seizure activity may also be excitotoxic (Meldrum, 1991), and that the metabolic demands and regulations of excitotoxicity or seizure may not be comparable to physiological activity underlying learning and memory.

Astrocytes are also poised to be a source of lactate for neurons because in the hippocampus and cortex they have been shown to express the type 5 isoform of lactate dehydrogenase (LDH-5; Bittar et al., 1996), which favors the conversion of pyruvate into L-lactate (Cahn et al., 1962). Neurons, on the other hand, do not express this isoform (Bittar et al., 1996), although both neurons and astrocytes express LDH-1, which catalyzes formation of pyruvate from L-lactate (Cahn et al., 1962). Thus, the differential expression of these enzymes also supports the idea that lactate is produced by astrocytes and shuttled to neurons, where it is then metabolized. The selective expression of other enzymes such as 6-phosphofructo-2-kinase/fructose-2,6-bisphosphatase 3 (Pfkfb3) and the M2 form of Pyruvate kinase (PKM2) provides astrocytes, in contrast to neurons, with a high capacity for aerobic glycolysis leading to L-lactate production (for review, see Magistretti and Allaman, 2015). However, a recent study demonstrated that neurons in resting conditions express higher hexokinase mRNA and protein levels than astrocytes, suggesting that neurons could at least initiate glycolysis (Lundgaard et al., 2015).

**Figure 1:**
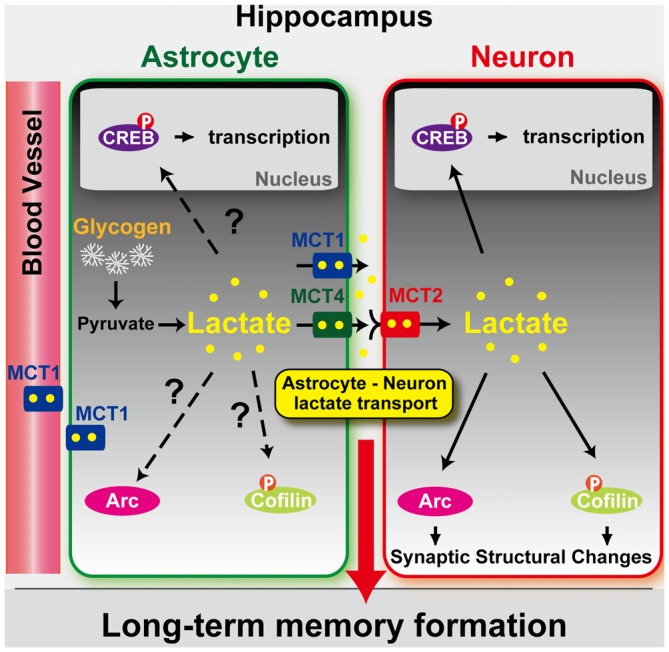
**Model depicting the role of astrocyte-derived lactate in long-term memory formation.** Astrocytic glycogen breakdown and lactate release are essential for long-term but not short-term memory formation, and for the maintenance of long-term potentiation (LTP) of synaptic strength elicited *in vivo*. Disrupting the expression of the astrocytic lactate transporters monocarboxylate transporter 4 (MCT4) or MCT1 causes amnesia, which, like LTP impairment, is rescued by L-lactate but not equicaloric concentration of glucose. Disrupting the expression of the neuronal lactate transporter MCT2 also leads to amnesia that is unaffected by either L-lactate or glucose, suggesting that lactate import into neurons is necessary for long-term memory. Glycogenolysis and astrocytic lactate transporters are also critical for the regulation of molecular changes required for long-term memory formation, including the induction of phospho-CREB, Arc, and phospho-cofilin. Adapted from Suzuki et al. (2011).

L-lactate shuttles among cells through monocarboxylate transporters (MCTs), which bidirectionally transport molecules with one carboxylate group, in particular lactate, pyruvate and ketone bodies. The transport occurs via diffusional, saturable co-transport of H^+^ until equilibrium is reached. In the brain, MCT4 is mainly expressed by astrocytes, whereas MCT2 is mainly expressed by neurons (Pierre and Pellerin, 2005; Rinholm et al., 2011), and MCT1 is expressed in astrocytes, oligodendrocytes, and endothelial cells of blood vessels (Gerhart et al., 1997; Rinholm et al., 2011). These MCTs are found not only in the brain but also in several other tissues of many species throughout evolution. While the expression distribution of MCT1 suggests that this transporter has a special role in lactic acid oxidation, the localization of MCT4 suggests that this transporter is found where lactic acid efflux predominates. Finally, MCT2 has a tenfold higher affinity for substrates than MCT1 and MCT4 and is found in cells where rapid uptake at low substrate concentrations may be required, including the proximal kidney tubules, neurons and sperm tails (Halestrap and Price, 1999). In Figure 1 a graphical model of L-lactate shuttling between astrocytes and neurons via MCTs is depicted.

Overall the question of how glucose and glycogen metabolism is regulated in neurons and astrocytes in resting conditions as well as under different conditions of stimulation remains debated (Chih and Roberts, 2003; Schurr, 2014). As we mentioned before, in this review we will not discuss the debates referring to different conditions of stimulation, as these arguments have been extensively described. In this review, we intend to discuss the literature and recent findings, including ours, related to the metabolism of glycogen, glucose and lactate in synaptic plasticity and memory formation in physiological conditions. Concerning the debates, we suggest that different paradigms and conditions of stimulation likely reflect different states of metabolism regulation. For example the metabolic regulations associated with oxidative stress conditions (e.g., stroke or traumas leading to neurodegeneration) or excessive activation of glutamate receptors (e.g., seizure; Patel et al., 2014), which causes cell damage, are very likely not comparable to those of physiological learning events. Thus, disagreements in the field of glucose/glycogen metabolism regulation in the brain may reflect, and hence be reconciled, by taking into account the differential regulations of glucose/glycogen metabolism in different stimulation conditions.

## Glycogen, Lactate and Astrocytic-Neuronal Monocarboxylate Transport in Plasticity and Memory

The use of lactate to metabolically couple astrocytes and neurons during high energy-stimulus-dependent demands, like the formation of persistent modifications in the brain after learning, which is at the basis of long-term memory consolidation and storage, is very intriguing because it could offer an explanation for concerted actions that occur at brain circuitry and whole system levels. This mechanism may represent a key regulation of memory processes for at least two reasons. First, memory consolidation and storage have high-energy demands, especially when they take place in conditions of arousal or stress, a critical regulator of long-term memories. Second, like other tissues that have high-energy functional requirements, the brain stores glucose in the form of glycogen, and does so mostly in astrocytes but not in neurons (Brown, 2004; Vilchez et al., 2007; Magistretti, 2008). Notably, it was reported in cell culture that breakdown of glycogen stores via glycogenolysis is associated with release of L-lactate but not glucose, suggesting that glycogen is functionally a lactate reservoir (Dringen et al., 1993).

As many studies that identified and characterized the role of lactate in astrocyte-neuronal coupling have been done in cell culture, conclusions for *in vivo* functioning in learning and memory needed *in vivo* validation. The first evidence for an important role of *in vivo* glycogenolysis in memory formation was the finding that glycogen stores diminish 30 min after taste aversion training in day-old chicks (O’Dowd et al., 1994; Hertz et al., 2003). 1,4-dideoxy-1,4-imino-D-arabinitol (DAB), an inhibitor of glycogen phosphorylation and breakdown, impaired taste aversion memory in day-old chicks when infused into the multimodal forebrain association region intermediate medial mesopallium (IMM), known to be required for memory consolidation (Gibbs et al., 2006). In this case glutamine was sufficient to rescue memory, presumably by contributing to a rescue of the glutamate/glutamine shuttle, which may also be affected by DAB. A subsequent study demonstrated that L-lactate was sufficient to rescue memory in the chick taste aversion model, both in the presence of DAB or 2-deoxyglucose, which impairs glucose phosphorylation and glycolysis (Gibbs et al., 2007). Notably, administration of D-lactate, the non-biologically active form, impaired chick taste aversion memory with a time delay that suggested it was inhibiting L-lactate metabolism and not uptake (Gibbs and Hertz, 2008).

Inspired by these studies, a few years ago we became interested in determining whether astrocytic mechanisms contribute to long-term memory formation *in vivo*, and specifically to hippocampal consolidation of a long-term episodic, aversive memory, using inhibitory avoidance (IA) in rats, a paradigm we had been studying for several years. IA is a long-lasting memory evoked by a single training trial, in which the animal associates the experience of a footshock with a given context upon entering it, and thus avoids it in future presentations. This memory requires intact hippocampus and amygdala, and has been extensively investigated in studies of arousal and stress-dependent modulation (Izquierdo et al., 1997; Roozendaal and McGaugh, 2011). IA models a behavioral response of an episodic aversive or stressful memory: its hippocampus-dependence makes it a useful model of emotional complex aversive memories, and its amygdala-dependence makes it an interesting model of emotional regulation of long-term hippocampal memory. Furthermore, this memory undergoes hippocampal-cortical system consolidation, a process that is typical of episodic and explicit memories, (those that in humans include declarative and autobiographical). In fact, in addition to the gene expression-dependent phase necessary for cellular consolidation, complex hippocampus-dependent memories undergo another consolidation process, which initially requires the role of the hippocampus, but with time becomes hippocampal-independent, while it maintains the engagement of cortical brain areas for memory storage and processing (Squire, 2004; Frankland and Bontempi, 2005).

**Figure 2:**
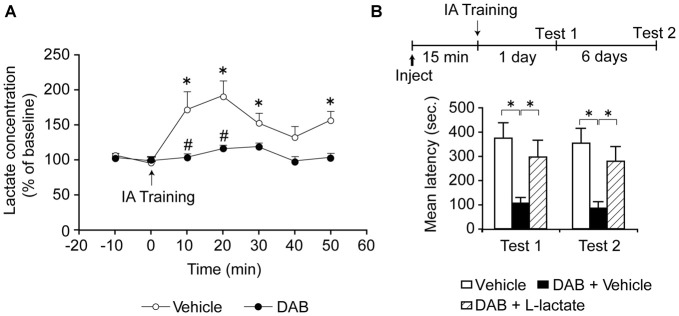
**(A)** Extracellular lactate levels expressed as % of baseline ± SEM (mean of the first two samples set 100%) within the dorsal hippocampus of freely moving rats measured by microdialysis. Rats were injected bilaterally into the dorsal hippocampi with either vehicle or 1,4-dideoxy-1,4-imino-D-arabinitol (DAB, 300 pmol). Dialysate fractions were collected 10 min prior to training (0 min, ↑) to determine baseline levels and collection then continued every 10 min for 50 min. Training significantly increased lactate levels over baseline (*p < 0.05) and this effect was abolished by DAB (^#^*p* < 0.05; *n* = 4/group). **(B)** Long-term memory retention expressed as mean latency values ± SEM in seconds (s) tested 24 h (Test 1) as well as 7 days (Test 2) after training. Vehicle, DAB (300 pmol), or DAB (300 pmol) + L-lactate (100 nmol) was administered bilaterally into the dorsal hippocampus 15 min prior to inhibitory avoidance (IA) training (*p < 0.05; *n* = 12/group). Adapted from Suzuki et al. (2011).

Focusing on the dorsal hippocampus, because this region is key for contextual associative learning, we found that training leads to an increase in extracellular L-lactate, as measured by *in vivo* microdialysis. This increase in lactate lasted at least 50 min after training, and was completely blocked by inhibition of glycogenolysis with DAB (Suzuki et al., 2011; Figure 2A). DAB also blocked long-term—but not short-term—IA memory indicating that glycogenolysis in the dorsal hippocampus is required for long-term memory consolidation (Figure 2B).

Similar results also done in rats were reported by Newman et al. (2011) on a different learning paradigm, spontaneous alternation, a spatial working memory task that requires the ventral hippocampus, and recently by Zhang et al. (2015a) and Boury-Jamot et al. (2015) in both cocaine-induced conditioned place preference (CPP) and self-administration consolidation or reconsolidation. Reconsolidation is a process of stabilization that occurs after memory retrieval and shares many similarities with consolidation (Alberini, 2005).

Studies in rodents had revealed that extracellular glucose levels in the hippocampus are depleted when rats learn and memorize tasks such as on a four-arm spontaneous alternation maze (McNay and Gold, 2002; Gold, 2014). Following up on these results, Newman et al. (2011) showed that extracellular L-lactate rapidly increases in the hippocampus with spontaneous alternation, while glucose decreases. The levels were determined using L-lactate and glucose-sensitive biosensors that measure current from a probe coated with lactate or glucose oxidase, respectively, and interestingly, allowed for temporal resolution of lactate and glucose concentration on the order of seconds. This temporal resolution revealed that the lactate increase slightly precedes the decrease in glucose, suggesting that the astrocytic responses may anticipate energy needs rather than responding to them (Newman et al., 2011). DAB also impaired memory for this task, and the impairment was rescued by ventral hippocampal injection of L-lactate, but, contrary to what we found with IA in the dorsal hippocampus, the memory impairment was also rescued by an equicaloric concentration of glucose, suggesting that, in this case, glucose was equivalent to L-lactate.

**Figure 3:**
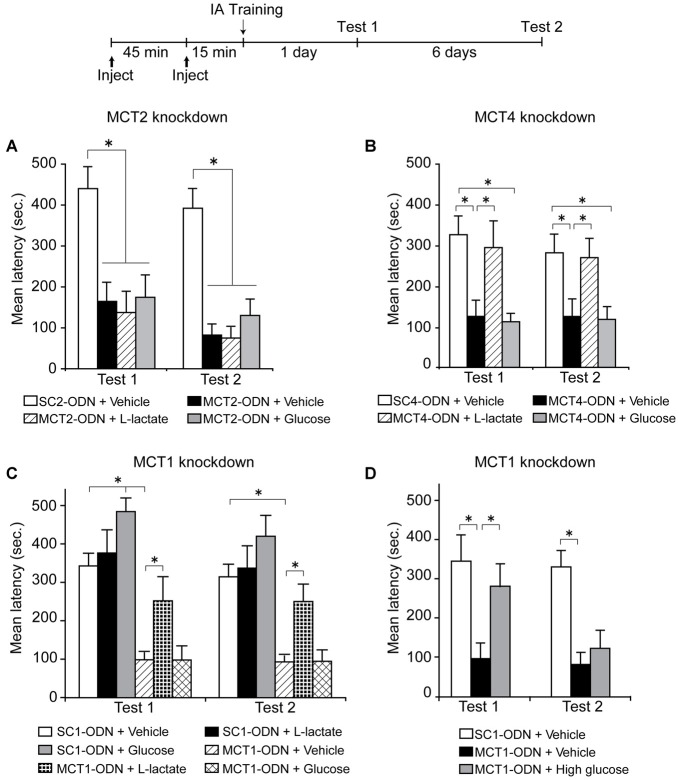
**Long-term memory retention expressed as mean latency values ± SEM in seconds (s) tested 24 h (Test 1) as well as 7 days (Test 2) after training. (A)** Dorsal hippocampi were injected bilaterally with scrambled MCT2 ODN (SC2-ODN; 2 nmol) 1 h before training + Vehicle 15 min before training, or antisense MCT2 ODN (MCT2-ODN; 2 nmol) 1 h before training + Vehicle, L-Lactate (100 nmol) or Glucose (50 nmol) 15 min before training (*n* = 6–8 per group). **(B)** Dorsal hippocampi were injected bilaterally with scrambled MCT4 ODN (SC4-ODN; 2 nmol) 1 h before training + Vehicle 15 min before training, or antisense MCT4 ODN (MCT4-ODN; 2 nmol) 1 h before training + Vehicle, L-Lactate (100 nmol) or Glucose (50 nmol) 15 min before training (*n* = 10–12/group). **(C)** Dorsal hippocampi were injected bilaterally with scrambled MCT1 ODN (SC1-ODN; 2 nmol) 1 h before training + Vehicle, L-Lactate (100 nmol) or Glucose (50 nmol) 15 min before training, or antisense MCT1 ODN (MCT1-ODN; 2 nmol) 1 h before training + Vehicle, L-Lactate (100 nmol) or Glucose (50 nmol) 15 min before training (*n* = 7–13/group). **(D)** Dorsal hippocampi were injected bilaterally with scrambled MCT1 ODN (SC1-ODN; 2 nmol) 1 h before training + Vehicle 15 min before training, or antisense MCT1 ODN (MCT1-ODN; 2 nmol) 1 h before training + Vehicle or High glucose (150 nmol) 15 min before training (*n* = 8–11/group). *p < 0.05. Adapted from Suzuki et al. (2011).

A subsequent experiment done both in our laboratory with IA and by Newman et al. (2011), asked whether L-lactate transport into neurons is necessary for memory formation, a critical prediction for the ANLS model. Using different approaches and tasks we both first tested whether MCT2 was necessary for memory formation. The conclusion from both studies was affirmative. MCT2 blocked either pharmacologically with α-cyano-4-hydroxycinnamate (4-CIN; Newman et al., 2011) or genetic targeting with antisense oligodeoxynucleotides (Suzuki et al., 2011) caused a significant and profound impairment in memory retention. Second, we, as well as Newman et al. (2011), asked whether L-lactate or glucose could rescue the amnesia. These experiments were addressing two important alternative explanations: the first was whether there was a critical flow of lactate in the opposite direction, from neurons to other cells such as astrocytes, as proposed by the neuronal-astrocytic lactate shuttle (Simpson et al., 2007; Mangia et al., 2009). If L-lactate exported via neuronal MCT2 played a critical role in these other cells contributing to memory consolidation, then the amnesia would be rescued by lactate and/or glucose. The second question was whether glucose entering into neurons was a preferential substrate for long-term memory formation. In this case, as glucose import into neurons is not stopped by the MCT2 blockade, the administration of glucose should have resulted in memory recovery. We both found that the amnesia caused by blocking MCT2 persisted in the presence of either lactate or glucose. Thus, these findings provided strong support that L-lactate transported into neurons via MCT2 is necessary for memory formation (Figure 3A).

In addition, when we blocked translation of MCT1 or MCT4 in the hippocampus using antisense oligodeoxynucleotides, which led to selective knock down of the respective protein, we found a blockade of long-term IA memory, as well as of the molecular cascades underlying long-term memory formation, indicating, again in agreement with the ANLS hypothesis, that the function of these transporters is necessary for memory formation. The memory deficit was in this case rescued by exogenous administration of L-lactate, but not of equicaloric glucose (Figures 3B,C). Moreover, with MCT1 knockdown, equicaloric concentration of glucose had no effect on memory impairment; however high concentrations of glucose (three times higher than equicaloric) rescued the memory loss, but the effect was transient (Figure 3D). While dorsal hippocampal administration of glucose led to a transient memory enhancement, administration of L-lactate did not change long-term memory retention compared to vehicle-injected controls (Figure 3C). From all these results, we concluded that the lactate produced by glycogenolysis, and therefore exported from astrocytes, must be imported into neurons in order to produce long-term memory formation, while an equicaloric concentration of glucose administration is not able to replace the effect of lactate.

We also found that astrocytic glycogenolysis is required for the maintenance of LTP at the Shaffer Collateral-CA1 synapse *in vivo* (Suzuki et al., 2011). In the presence of DAB, which did not affect baseline neurotransmission, tetanic stimulation produced a strong initial potentiation, which, however, rapidly decayed. Co-injection of L-lactate with DAB before LTP induction reversed this decay.

Together, all these data strongly supported the conclusion that lactate-mediated mechanisms and lactate transport from astrocytes into neurons are essential for long-term plasticity and long-term memory consolidation.

Some alternate explanations of our results have been offered in a recent review by Dienel (2015). One question raised is that the results should be considered in the context of noradrenaline (NA) release from locus coeruleus projections to the hippocampus and the multiple regulations that this would target. Indeed, as also detailed below, the role of NA and its receptors in memory formation and modulation as well as stimulation of astrocytic glycogenolysis is well documented (Sorg and Magistretti, 1991; Gibbs and Summers, 2002; McGaugh and Roozendaal, 2002). IA has actually been extensively investigated as a model of memory modulation by arousal and stress (Gold and Van Buskirk, 1975; McGaugh and Roozendaal, 2002). We in fact agree that NA may act through astrocytic metabolic mechanisms. Ongoing studies in our laboratory investigating how NA as well as β adrenergic receptors (βARs) expressed by hippocampal astrocytes play a critical role in memory formation suggest that they function by engaging the ANLS. We will return to this point below.

A second issue raised is that the dose of L-lactate used for memory rescue in our study (Suzuki et al., 2011) is excessive and possibly pathophysiological and may suppress neuronal firing. However, the concentrations of L-lactate we used were within range of physiological levels (approximately 5 mM), and did not elicit any tissue damage or altered response such as seizure. Similar concentrations of L-lactate used by Newman et al. (2011) also enhanced physiological responses such as spontaneous alternation memory, again excluding a toxic effect. Finally, a similar concentration of L-lactate with DAB in *in vivo* electrophysiology experiments did not preclude LTP induction, which also argues against a suppression of neuronal firing.

Lastly, it was indicated that: “*lactate transport was not measured* and *shuttling was assumed to occur*.” Unfortunately techniques that directly detect the flux of lactate are still missing. However, our results based on transport blockers and directional rescuing experiments, in agreement with evidence from cell culture studies and slice preparations, are consistent with the conclusion that lactate is transported from astrocytes to neurons. Astrocytes can generate and release lactate (reviewed in Pellerin et al., 2007), which neurons are capable of utilizing as an energy source (Schurr et al., 1988; Izumi et al., 1997) while at the same time they appear to have a lower enzymatic capacity to generate lactate (Bittar et al., 1996). In agreement, a recent study *in vivo* in the anesthetized mouse reported evidence for a lactate gradient from astrocytes to neurons (Mächler et al., 2016). Hence, collectively our results, in line with others, indicate that lactate released from astrocytes and entered into neurons is critical for memory consolidation. Nevertheless, as other substrates such as pyruvate and ketone bodies can be transported via MCTs (Poole and Halestrap, 1993), it cannot be excluded that they may also critically contribute to memory consolidation.

Recently, Zhang et al. (2015a) and Boury-Jamot et al. (2015) repeated the experimental design we carried out in Suzuki et al. (2011) to ask whether glycogenolysis, lactate release and MCT1, 4 and 2 in the amygdala play a critical role in the consolidation and/or reconsolidation of cocaine-induced CPP or self-administration, and found that to be the case (Zhang et al., 2015a). Furthermore, Tadi et al. (2015) using IA in mice found that mRNA levels of MCT1, MCT4, the α2 subunit of the Na/K-ATPase and glucose transporter type 1 increase at 24 h following IA training. They also showed that mice heterozygous for an MCT1 targeted disruption have impaired IA memory, again supporting the conclusion that genes involved in glia metabolism play a critical role in memory formation.

An important additional mechanism was recently described (Sotelo-Hitschfeld et al., 2015), by which L-lactate can be released in the interstitial space in response to cell depolarization through permeable ion channels, which suggests a neuromodulatory and gliotransmitter role of lactate. The identification of the channel/s will allow for testing whether this release also plays a role in learning and memory.

Indeed, L-lactate may not only be an energy substrate but may provide additional important regulations: a number of recent studies suggest that lactate may be critical because in addition to providing energy it can: (1) act as a transmitter of metabolic information by modulating prostaglandin action and cerebral vasodilatation causing cerebral blood flow to increase; (2) regulate the NADH/NAD+ redox ratio by conversion to pyruvate; and finally (3) activate the G-protein-coupled receptor GPR81 (also known as hydroxycarboxylic acid receptor 1) in astrocytes, capillaries and neurons which inhibits cAMP production (Gordon et al., 2008; Bergersen and Gjedde, 2012; Lauritzen et al., 2014). Thus, lactate physiologically produced by astrocytes may play multiple functions including providing energy, function as a signaling and/or modulatory molecule (Barros, 2013), and act as a preferential substrate, because it might spare glucose for other more specific uses such as glutamate synthesis (Bouzier-Sore et al., 2003).

All these functions may be critically involved in parallel, or regulated in different conditions and/or stages of synaptic plasticity and memory formation, and the field is now in a position to test all these hypotheses.

## The Functions of Lactate Transported Into Activated Neurons for Memory formation: Translation and Molecular Changes induced by Learning

What is the function of lactate entering activated neurons? What does lactate provide, once inside the neurons that is critical for long-term memory formation? Is this mechanism general for different tasks, brain areas and memory modulations?

First, as lactate is a glycolytic metabolic substrate, an obvious question is whether it supplies energy. If that is the case, the next question is: what is this energy necessary for in long-term memory consolidation? Astrocytes are spatially well placed to couple neuronal activity with neuronal metabolic needs because they have processes that enwrap synapses as well as endfeet that enwrap blood vessels (Belanger et al., 2011). The shape and function of neurons suggests that when they are under high energy consumption, such as with excitatory postsynaptic potentials, the oxidation of glucose present in the neuron may not be sufficient, and an additional source of energy is needed; astrocytes have the structure and ability and have been shown to function precisely as an energy storage and supplier for neurons when they are in high energy demands. We refer to several excellent reviews that provide extensive descriptions of this matter (Magistretti, 2006; Belanger et al., 2011; Barros, 2013).

To begin addressing the question of the critical role of lactate in long-term memory formation, we reasoned that, first, the energy generated by lactate may be necessary for high-energy demanding functions required to form long-term memories; for example *de novo* transcription and translation, as well as the resulting structural modification of synapses. Translation occurs at activated synapses, where the process of gene expression regulation critical for memory formation seems in fact to rapidly start with activity (Martin et al., 2000). Thus, we have asked whether fundamental molecular changes required for long-term synaptic plasticity and memory formation are dependent upon lactate released by training. It is well established, in many species and different types of memories, that consolidation requires the activation of gene cascades, including that mediated by the transcription factor cAMP response element binding protein (CREB), which plays an evolutionarily conserved role in memory formation (Alberini, 2009). Additional critical mechanisms of long-term memory formation include the activation of the actin severing protein p21-activated kinase-cofilin (Chen et al., 2007), as well as the induction of the immediate early gene activity-regulated cytoskeletal protein (Arc/Arg3.1), both of which are key regulators of structural changes at synapses (Fischer et al., 2004; Chen et al., 2007; Bramham et al., 2008; Mantzur et al., 2009). Intra-dorsal hippocampal administration of DAB did not affect baseline levels of these markers in untrained rats, but completely blocked the activation of CREB and cofilin as well as the induction of Arc in rats trained in IA. These changes were reversed by co-administration of L-lactate (Suzuki et al., 2011). Notably, Arc is a neuronal protein (Vazdarjanova et al., 2006); thus our data indicated that neuronal molecular mechanisms occurring following learning depend upon glycogenolysis and lactate release.

In agreement with the conclusion that lactate controls molecular modifications in neurons, Yang et al. (2014) reported that administration of L-lactate to primary neuronal cortical cultures, or into mouse sensory-motor cortex, induces the expression of Arc along with the other immediate early genes c-Fos and Zif268. Notably, these changes are mediated by N-methyl-D-aspartate receptor (NMDAR) activation, and the engagement of the extracellular-signal-regulated kinase 1/2 (ERK1/2) signaling, indicating that they are activity-dependent.

Lactate may also have concerted actions as a signaling molecule. In the mitochondria, lactate is oxidized to pyruvate while regenerating NADH, thus affecting the cellular and organelle redox state. In cultures, application of lactate is associated with increased intracellular levels of NADH, and application of NADH alone induces similar effects as lactate on NMDAR signaling (Yang et al., 2014). Therefore, lactate transport may serve as a signaling mechanism between cells and intracellularly via changes in redox balance (Brooks, 2009). For example, lactate oxidation can directly activate reactive oxygen species (ROS) production, and although excessive ROS production leads to oxidative damage, physiological levels of ROS are required for synaptic plasticity (Massaad and Klann, 2011). Thus, further studies should investigate whether lactate functions also as a regulator of redox balance and of signaling in models of memory formation. Lactate in fact is also known to act at GPR81, which is expressed on post-synaptic membranes of excitatory synapses, and the activation of this receptor is known to reduce cAMP levels (Gundersen et al., 2015; Morland et al., 2015). However, a role for GPR81 in synaptic plasticity and memory has not been investigated, and further studies are necessary to understand this role.

Collectively, these results suggest that the metabolic functions of lactate may be needed to supply the necessary energy required for cellular mechanisms (including neuronal mechanisms) evoked by training and critical for processing memory consolidation. This distinctive role of lactate may actually be an important differential mechanism that gates the changes necessary to make and maintain long-lasting structural modification, as opposed to feeding the preservation of survival or homeostatic mechanisms of cells. We suggest that when activity-dependent changes involving high-energy demands occur, such as those required for episodic long-term memory consolidation in the hippocampus, glycogenolysis and glycolysis leading to lactate production by astrocytes is a necessary step that allows the molecular and morphological modifications underlying memory storage to occur. We also suggest that, consistent with the results found in cell cultures, additional regulatory mechanisms such as redox regulation and signaling are likely to be activated by lactate, and that lactate may not be solely a resource for energy in memory formation. We speculate that the coordinated action of all lactate-mediated changes may be necessary for the multiple cellular and system effects that lead to the long-lasting biological changes necessary for memory storage.

## The Functions of Lactate in Memory formation: Glutamine/Glutamate Shuttle

In addition to providing an energy source for molecular regulations, lactate coupling between neurons and astrocytes may function to regulate and monitor glutamate-glutamine turnover. Glutamatergic neural activity underlies formation of LTP (Milner et al., 1998), and is therefore essential for memory consolidation. Given that glutamate activity is believed to drive the ANLS (Pellerin et al., 2007) it appears the neurotransmitter contributes to both providing a signal for the production of lactate and as a mechanistic target of lactate. Astrocytes participate in the glutamate-glutamine shuttle wherein they take up glutamate from the active synapse and convert it into glutamine, which is then transported back to neurons to be metabolized back into glutamate (Gibbs et al., 2008b). This cycle uses the same principles of metabolic compartmentalization as the ANLS, given that astrocytes, but not neurons, express glutamine synthetase with which to metabolize glutamate (Norenberg and Martinez-Hernandez, 1979). A model has been proposed which postulates that cotransport of glutamate and Na^+^ into astrocytes results in enhanced activity of the Na^+^-K^+^ ATPase and associated generation of lactate via aerobic glycolysis (Magistretti, 2009). During periods of elevated ammonium levels in the brain, which can lead to memory impairment (Aguilar et al., 2000; Muñoz et al., 2000), both lactate levels and glutamine synthetase activity are increased, illustrating a codisruption of the astrocyte neuronal coupling cycles under metabolic insult. Lactate generation essentially tracks glutamatergic activity and could potentially act as a signal for the rate at which astrocytes must import synaptic glutamate for purposes of recycling it and preventing excitotoxicity. Additionally, rather than be converted into glutamine, glutamate can also be directed toward the citric acid cycle (Gibbs et al., 2008b). Interestingly, cultured cortical astrocytes supplied with 0.2–0.5 mM glutamate converted a portion of the glutamate into lactate, which was released into the culture media (McKenna et al., 1996). We have not tested the effect of glutamine with DAB in our IA paradigm, but our data do not exclude that this may in fact function in parallel with the lactate-dependent changes we have reported (Suzuki et al., 2011). Taken together the glutamate-glutamine shuttle and the ANLS may be two subcomponents of a broad metabolic coupling between neurons and astrocytes, which have significant importance in memory consolidation.

## The Functions of Lactate in Memory formation: Stress, Neuromodulation, Noradrenaline and Glucocorticoids

Another domain with important ramifications for memory formation and modulation, where the ANLS may play a significant role, is stress. Stress affects all aspects of physiology, including brain functions, and hence, memory formation, retrieval and storage. In humans, as in animal models, a large body of literature has shown that, for complex memories such as those processed by the hippocampal-dependent system, the degree of stress and the degree of memory retention are related by an inverted-U function (de Kloet et al., 2005). Accordingly, the stress hormones NA and glucocorticoids modulate memory retention: low concentrations of these hormones enhance memory while high concentrations impair memory retention (de Kloet et al., 2005; Andreano and Cahill, 2006; Roozendaal and McGaugh, 2011; Herman, 2013).

Blocking ARs in the amygdala blocks IA memory enhancement by stress (Liang et al., 1986) or by glucocorticoid receptors (GRs) agonism (Quirarte et al., 1997). While the memory enhancing effects of NA in the basolateral amygdala have been more extensively investigated, less is known about the effects of NA in the hippocampus, despite projections from the locus coeruleus releasing NA in the hippocampus where it directly modulates hippocampal-dependent memories (Sara, 2009). As mentioned before, the hippocampus is critical for episodic, explicit and declarative memory formation, and engages in crosstalk with the amygdala to encode and store emotionally relevant information (McGaugh, 2013). This crosstalk has been suggested by evidence of the synchronized theta activity found in both the amygdala and hippocampus after fear conditioning (Paré et al., 2002; Seidenbecher et al., 2003; Narayanan et al., 2007), as well as by links in codependent molecular responses. For example, Arc increases in the dorsal hippocampus following injections of β_2_ adrenergic agonists into the basolateral amygdala (McIntyre et al., 2005). Moreover, studies directly targeting the hippocampus showed that stress mechanisms in this region change emotional memories: infusion of NA into the dorsal hippocampus increases contextual fear learning (Yang and Liang, 2014), and in fact regulates hippocampal molecular mechanisms critical for long-term plasticity, such as CREB activation (Kabitzke et al., 2011). The NA effect on the hippocampus is mediated by βARs, as the antagonist propranolol given either systemically or into the dorsal hippocampus blocks contextual fear memories including IA, as well as spatial memories (Stuchlik et al., 2009; Kabitzke et al., 2011; Chen et al., 2012). This suggests that not only the amygdala but also the hippocampus is a main target of stress regulation. This is in line with the large body of literature showing that chronic stress suppresses dentate gyrus neurogenesis and causes dendrites of hippocampal (and medial prefrontal cortical) neurons to shrink, whereas it causes basolateral amygdala neurons to increase in dendritic complexity and sprout new synapses (Miller and McEwen, 2006). Thus, the NA-mediated role in memory consolidation or enhancement, through the action of amygdala and/or hippocampus, may be differentially regulated by distinct cell types, and therefore by distinct NA receptor subtypes (Gibbs et al., 2008c; Hutchinson et al., 2008; McReynolds et al., 2010). Further studies are needed to address this important issue.

As detailed below, there is evidence suggesting that stress regulates lactate formation and in turn, lactate seems to be critically involved in regulating responses to stress. We suggest that the hippocampal ANLS may participate in both adaptive and maladaptive stress-mediated allostasis. When arousal or stress occurs, the release of adrenaline from adrenal glands produces changes that are necessary for the organism to adapt to the new condition: one of these changes is the breakdown of glycogen stores in the liver, which leads to an increase in blood glucose levels. This glucose is a source of energy for all the necessary responses. The peripheral increase in adrenaline is paralleled by a release of NA and glucocorticoids in the brain, the stress hormones that are required for memory consolidation, as well as its modulation (McGaugh and Roozendaal, 2009; Chen et al., 2012). Notably, both NA and glucocorticoids regulate the metabolism of energy substrates necessary to brain cells for processing the experience to be remembered. Thus, a rise in blood glucose subsequent to adrenaline may mediate, at least in part, the effects of the hormone on memory (Gold, 1995).

It has been suggested that the increased glucose metabolism after stress may take place in the brain, like in the liver, through the recruitment of glycogenolysis (Allaman et al., 2004). In the brain glycogenolysis occurs in astrocytes, which would therefore provide lactate as an energy substrate to neurons. In other words, it is possible that stress increases lactate release in the brain through astrocytic glycogenolysis and glycolysis. In agreement with this idea, restraint stress (De Bruin et al., 1990; Elekes et al., 1996) and electric shock (Krugers et al., 1992) stimulate intrahippocampal lactate formation, indicating that NA release and lactate formation during stress are indeed potentially linked. Furthermore, L-lactate seems to regulate NA availability because lactate released by activated astrocytes in the locus coeruleus leads to neuronal excitation and release of NA (Tang et al., 2014).

How do stress and lactate come together in processing memory consolidation? In pioneering studies performed in chicks, Gibbs and colleagues revealed that noradrenergic activation via both α and βARs is critical for memory processing through distinct effects on metabolism (Hourani et al., 1990; Gibbs et al., 2008b). As with mammals, a memory elicited by a weak training in chick can be enhanced by NA (Crowe et al., 1990; Gibbs, 1991). Infusion of the β_2_AR agonist zinterol or the β_3_AR agonist CL316243 into the intermediate hyperstriatum ventral/medial neostriatum (IMVH) of chicks enhances taste aversion memory consolidation, indicating that NA promotes memory consolidation via these receptors (Herman, 2013). Furthermore, different subtypes of βAR were found implicated in the effects of lower or higher doses of NA: subcutaneous administration of the β_1_ + β_2_AR antagonist propranolol interfered with NA-mediated memory consolidation at medium doses of NA infused into the IMHV, whereas the β_3_AR antagonist SR59230 impaired consolidation at the low doses (Herman, 2013). It was subsequently demonstrated that the memory enhancing effects of NA are achieved through metabolic mechanisms. β_2_AR appears well positioned to regulate lactate availability during memory formation. Subcutaneous administration of zinterol augmented memory consolidation in the chick taste aversion paradigm (Gibbs et al., 2008c), but this effect was mitigated by DAB injection into the IMM, suggesting that glycogenolysis underlies the memory enhancement. In agreement, DAB also prevented a zinterol-induced reduction in glycogen in cultured astrocytes (Gibbs et al., 2008a,c; Gibbs, 2008). In contrast, the memory enhancing effects of β_3_AR agonism were blocked by the glucose transport inhibiting compounds cytochalasin B, phloretin, or phlorizin (Gibbs et al., 2008c). Cytochalasin B inhibits GLUT 1, a glucose transporter found on astrocytes and endothelial cells, whereas phloretin is a general GLUT inhibitor and phlorezin inhibits Na^+^/energy-dependent endothelial glucose transport (Gibbs et al., 2008c). Whether or not β_3_AR mediated uptake of glucose by astrocytes serves to provide these glial cells with a substrate for lactate production or for glycogen synthesis is an intriguing question. The same pattern of β_2_AR playing a role in glycogenolysis and β_3_AR supporting glucose uptake was found in the avian hippocampus (Gibbs et al., 2008a).

Prior to the work in cultured chick astrocytes, NA had been found to stimulate glycogenolysis in cultured mammalian cortical cells (Magistretti and Morrison, 1988). This effect was blocked by βAR as well as α_2_AR antagonism (Subbarao and Hertz, 1990). Antagonism of α_1_AR had no effect, which is consistent with behavioral impairment of taste aversion memory via α1AR (Subbarao and Hertz, 1990). Hence, it is possible that excess levels of NA under very stressful circumstances could impair memory consolidation through α_1_AR-mediated inhibition of glycogenolysis. We are suggesting that the degree of a stressor could potentially affect the strength of memory consolidation by regulating the levels of lactate available within the brain to support metabolism and plasticity. At higher levels of stress memory could be impaired due to dysregulation of lactate formation, which may then result in a dysregulation of its metabolism.

Glucocorticoids appear to synergize with NA in the mobilization of energy during a stress response (Joëls and Baram, 2009). Astrocytes express GRs (Bohn et al., 1994; Simard et al., 1999) and corticotrophin releasing hormone receptors (Kapcala and Dicke, 1992), and respond to glucocorticoids with calcium waves (Simard et al., 1999) and peptide release (Chatterjee and Sikdar, 2013). The breakdown of glycogen following NA stimulation is followed by a seemingly compensatory increase in rates of glycogenesis (Sorg and Magistretti, 1992), which is inhibited by dexamethasone (Allaman et al., 2004), a synthetic form of glucocorticoid (Sapolsky et al., 2000). It is tempting here to speculate that glucocorticoids as well as NA affect memory formation and modulation by acting through astrocytic functions and a critical one is the glycolytic pathway that provides lactate to neuronal functions.

Consequently, we can expect that in maladaptive conditions, for example prolonged or chronic stress, the lactate metabolic role may be significantly altered. In fact, it has been proposed that glycogen depletion following prolonged stress and glucocorticoid exposure could lead to pathological states of the brain (Allaman et al., 2004). In accordance with this hypothesis, mice chronically exposed to glucocorticoids exhibited reduced levels of hippocampal glycogen, which appeared to derive from reduced glycogenic and increased glycolytic activity (Zhang et al., 2015b). These changes corresponded with increased immobility in forced swim test and tail suspension test wherein high levels of immobility are taken to suggest depression-like behavior (Zhang et al., 2015b). Additionally, prolonged social defeat stress in tree shrews (*Tupaia belangeri*, Czeh et al., 2006) and rats (Araya-Callís et al., 2012) is associated with significantly reduced counts of astrocytes that stain for glial fibrillary acidic protein (GFAP), a glial cytoskeletal element. In tree shrews these astrocytes also exhibit a 20% reduction in somal volume (Czeh et al., 2006), implying that alteration of astrocytic function critically contributes to the effect of stress and of stress-induced psychopathologies.

Together, these results offer support for some intriguing hypotheses for conditions of high or prolonged stress, which as mentioned before, critically contribute to psychopathologies: the failure to adapt in the long term could result in glycogen depletion, hence a shortage of available fuels required for coping over the longer term. The hippocampus is especially sensitive to the deleterious effects of glucocorticoids, and is a principle target in the brain for glucocorticoid dysregulation (Sapolsky, 1990).

In sum, several lines of evidence report an important contribution of lactate in the brain in the regulation of stress hormone-mediated responses, including memory formation in adaptive conditions but also, possibly through distinct mechanisms, in acute high or prolonged stress.

## Conclusion

Work exploring the role of lactate in long-term memory formation supports the conclusion that memories that are formed after salient or stressful experiences critically engage glycogenolysis and glycolysis to support several mechanisms that lead to long-term morphological synapse modification. As glycogenolysis leading to lactate formation is a function of astrocytes, we suggest that the ANLS hypothesis provides important insights into the basic metabolic physiology that underlies the formation of memories. We speculate that the energy required for making long-term structural changes in synaptic networks is a metabolic signature of memory consolidation, and this is provided by lactate generation from astrocytes and its transfer into activated neurons. There remains much to be elucidated regarding how the ANLS may contribute to memory formation and processing under normal physiological conditions and then by extension, how it performs in pathological states. This knowledge will likely provide explanations and suggestions for new therapies targeting metabolic, stress and brain disorders.

## Author Contributions

CMA conceived and wrote the review; MQS and VG wrote the review.

## Conflict of Interest Statement

The authors declare that the research was conducted in the absence of any commercial or financial relationships that could be construed as a potential conflict of interest.
